# Supplementary material for: Identification and Evolutionary Analysis of Cotton (Gossypium hirsutum) WOX Family Genes and Their Potential Function in Somatic Embryogenesis
Source: Int J Mol Sci. 2023 Jul 4;24(13):11077. doi: 10.3390/ijms241311077 (PMC10342170; doi:10.3390/ijms241311077)
Supplement: Supplementary file 1 [file ijms-24-11077-s001.zip › Figure S1.pdf]

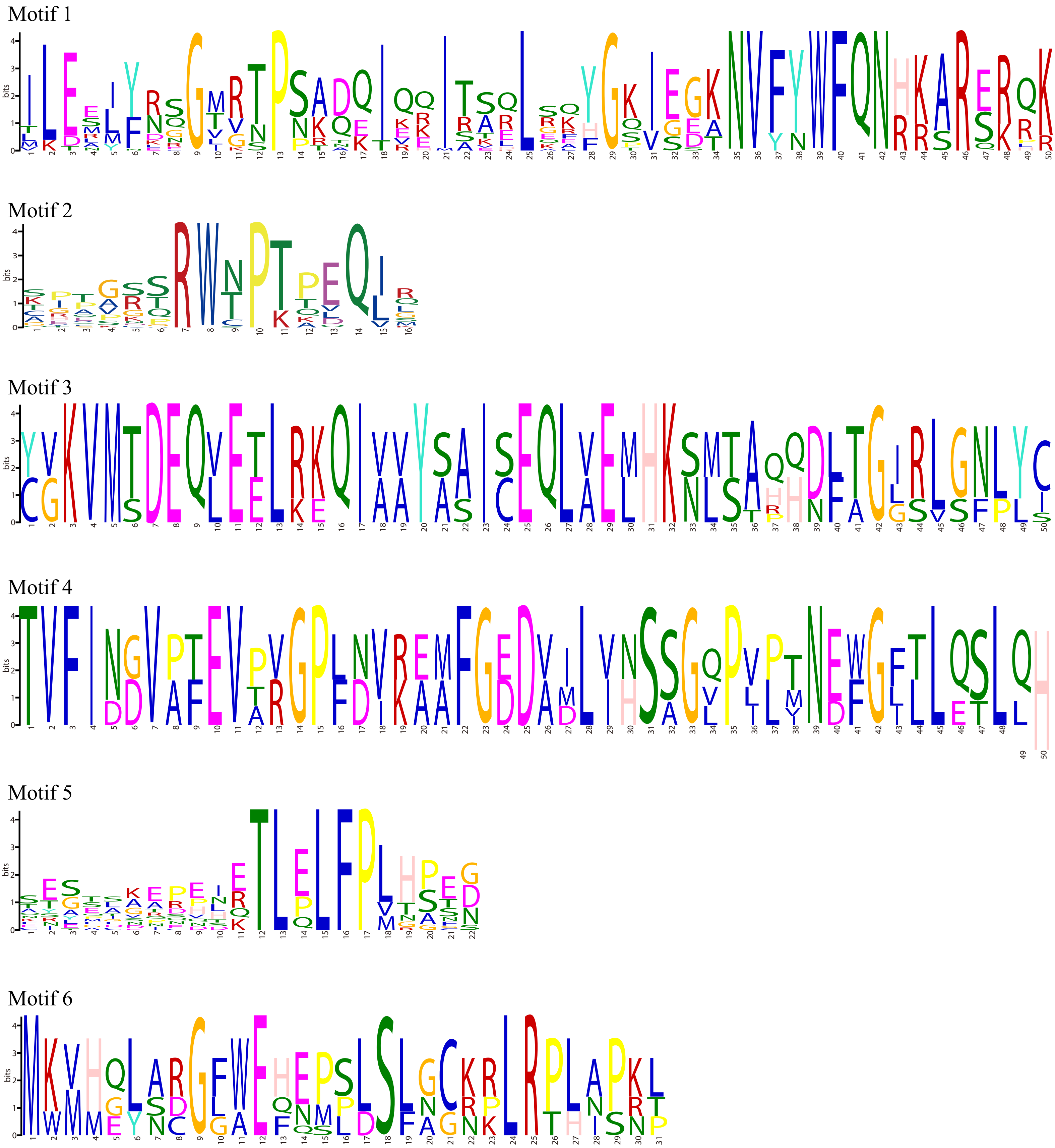

Figure S1. Sequence logos of six motifs indentified in cotton WOXs. The *x*-axis represents the relative positions of the motifs and *y*-axis represents the information contents as measured in bits. Motif 1-6 depict the different conserved motifs present in cotton WOX protein sequences.
