# Supplementary material for: Identification and Evolutionary Analysis of Cotton (Gossypium hirsutum) WOX Family Genes and Their Potential Function in Somatic Embryogenesis
Source: Int J Mol Sci. 2023 Jul 4;24(13):11077. doi: 10.3390/ijms241311077 (PMC10342170; doi:10.3390/ijms241311077)
Supplement: Supplementary file 1 [file ijms-24-11077-s001.zip › Figure S2.pdf]

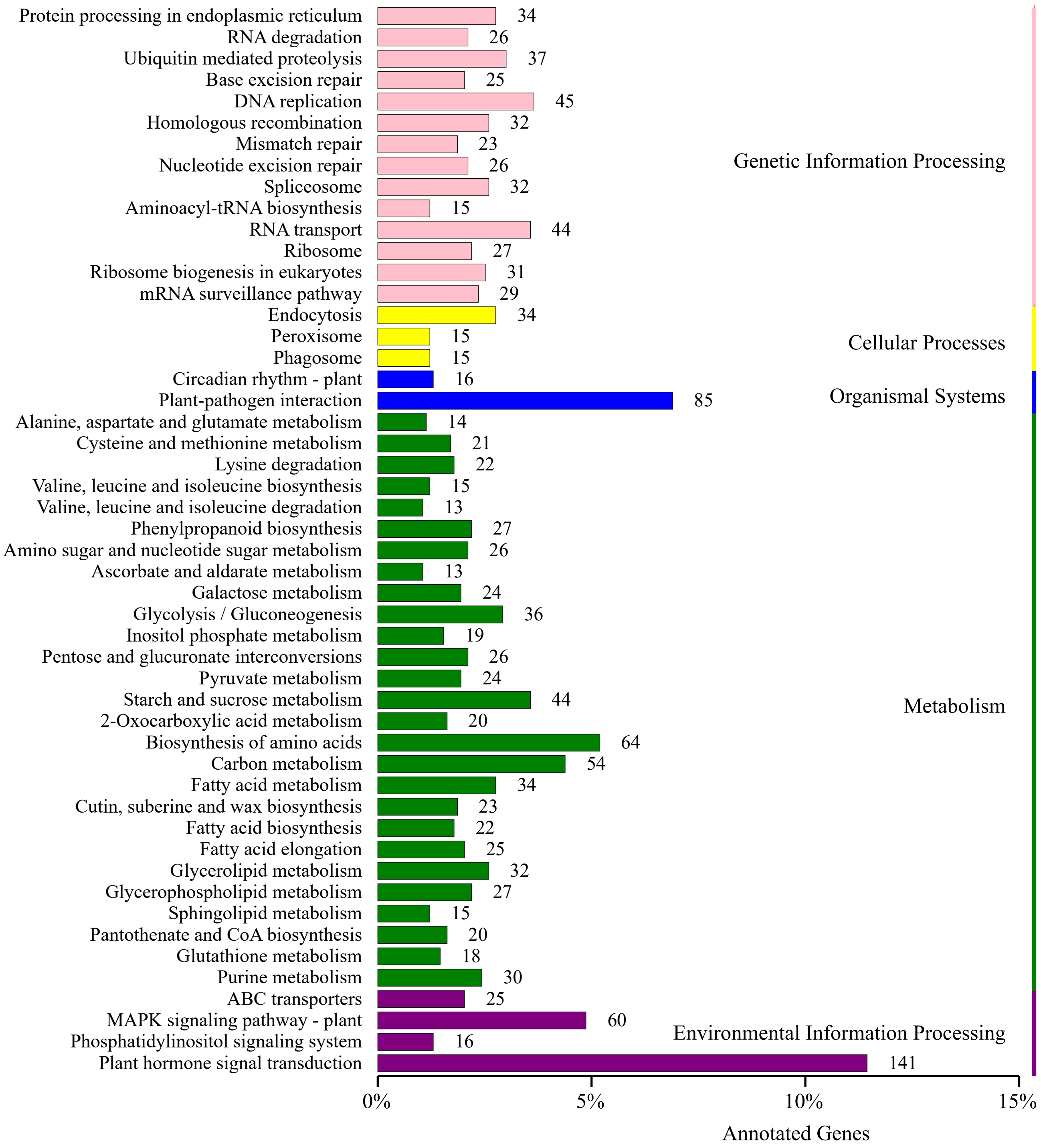

Figure S2. Summary of KEGG pathway annotations of genes co-expressed with WOX genes in the embryogenic tissues associated moduleduring somatic embryogenesis. The numbers next to the bar represent the number of genes annotated to the specific KEGG pathways.
