# Supplementary material for: Identification and Evolutionary Analysis of Cotton (Gossypium hirsutum) WOX Family Genes and Their Potential Function in Somatic Embryogenesis
Source: Int J Mol Sci. 2023 Jul 4;24(13):11077. doi: 10.3390/ijms241311077 (PMC10342170; doi:10.3390/ijms241311077)
Supplement: Supplementary file 1 [file ijms-24-11077-s001.zip › Figure S3.pdf]

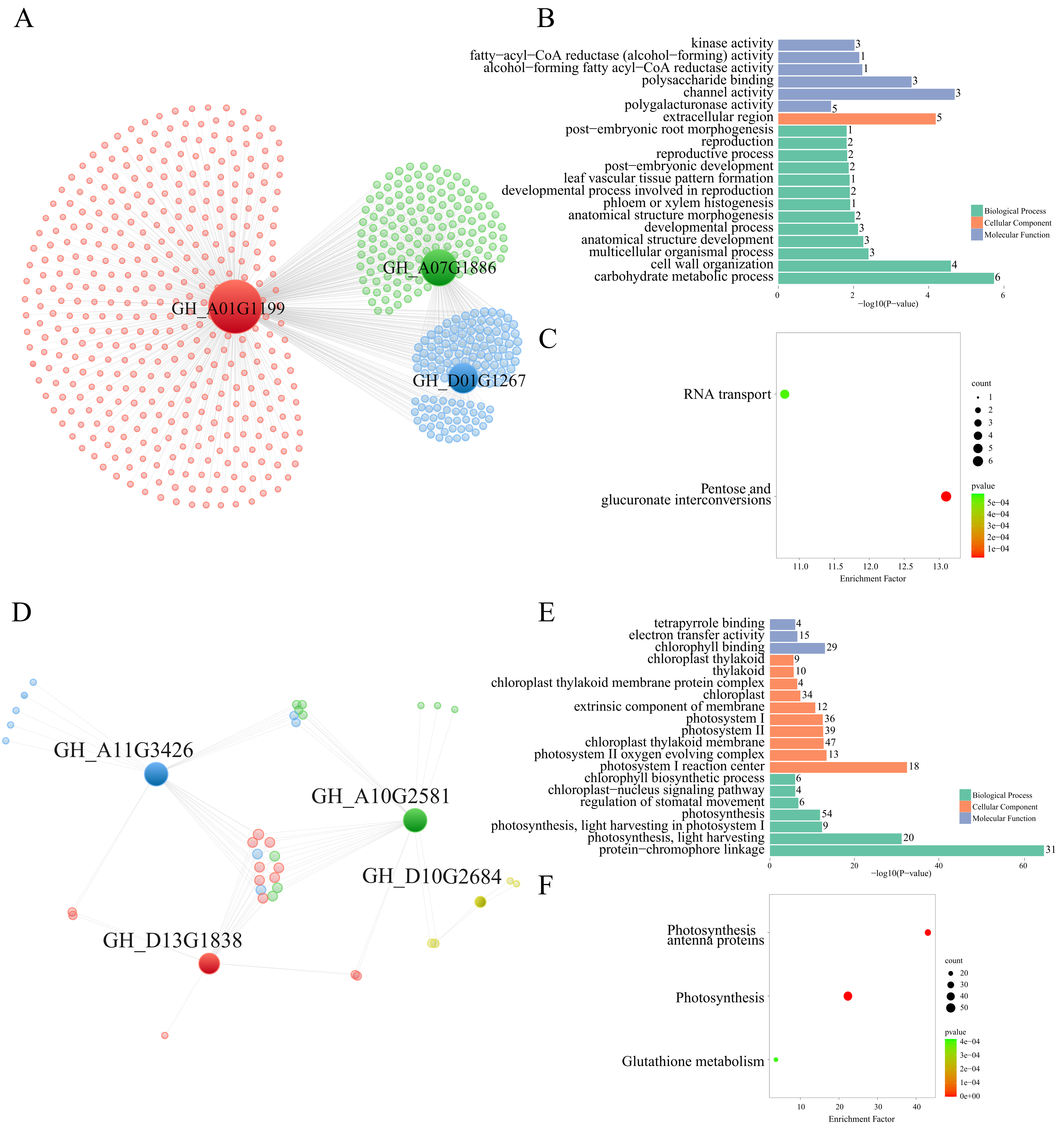

Figure S3. Co-expression network of cotton WOX genes during somatic embryogenesis based on the two other WGCNA resulted gene modules involving WOX genes that corresponding to the cluster I (**A**) and cluster III (**D**) of the above expression clustering heatmap. Genes co-expressed with cotton WOX genes are represented by circle nodes in different colors, lines linked two nodes represented co-expression relationships between them. The size of nodes represented the numbers of WOX genes that showed co-expression relationship with the specific gene. The GO term and KEGG pathway enrichment analysis results of genes co-expressed with cotton WOX genes of cluster I (**B** for GO term and **C** for KEGG pathway) and cluster III (**E** for GO term and **F** for KEGG pathway) were showed respectively.
