# Supplementary material for: Identification and Evolutionary Analysis of Cotton (Gossypium hirsutum) WOX Family Genes and Their Potential Function in Somatic Embryogenesis
Source: Int J Mol Sci. 2023 Jul 4;24(13):11077. doi: 10.3390/ijms241311077 (PMC10342170; doi:10.3390/ijms241311077)
Supplement: Supplementary file 1 [file ijms-24-11077-s001.zip › Table S1.pdf]

Table S1. Primers used in qRT-PCR

Gene ID	Primer F/R
GH_A02G2045	GACTCTCCGGAAGCAGATTGCC/ TTTGTGCCCACCCGATGTCATT
GH_D03G0016	TAAGTGCGAGACAACGGTGGAC/ CAGAAATGGGGCCATGCTGACT
GH_A05G1589	GCTCCTCCTATCACTAATATCTCCA/ GAGAACTGGAAACGTCAACGAC
GH_A05G2053	GTTACAGCAGCACACCTCCT/ GCGTAGGATTCCATCGCGTA
GH_D05G1617	TGCCAAAAAGCCCCTTACAC/ GGTTAACCATCCCGGAGTTGA
GH_A11G2763	GAAGCGTGTGGTTGTCATCG/ GGAGATCGTGGCTGAAAACG
GH_D11G2789	CAAGCGTAACCATGCACCTC/ ATTGCGATGACAACCACACG
GH_A09G0177	GTGAAACACCAACTCGCACC/ GGCTGCATGGTTCTTCAGAC
GH_D09G0172	GTGAAACACCAACTCGCACC/ AGGTTATGACGATGGGTGGC
